# Alarming prevalence and clustering of modifiable noncommunicable disease risk factors among adults in Bhutan: a nationwide cross-sectional community survey

**DOI:** 10.1186/s12889-017-4989-x

**Published:** 2017-12-21

**Authors:** Dorji Pelzom, Petros Isaakidis, Myo Minn Oo, Mongal Singh Gurung, Pemba Yangchen

**Affiliations:** 1 Health Management and Information System Unit, Policy and Planning Division, Ministry of Health, P.O. 726, Thimphu, Bhutan; 2 Médecins Sans Frontières, Operational Research Unit, Luxembourg City, Luxembourg; 3 International Union Against Tuberculosis and Lung Disease (The Union), Mandalay, Myanmar

**Keywords:** Operation research, SORT-IT, WHO STEPS, NCD

## Abstract

**Background:**

Bhutan is currently facing a double burden of non-communicable (NCDs) and communicable diseases, with rising trends of NCDs. The 2014 STEPS survey identified high prevalence of several NCD risk factors; however, associations with socio-demographic characteristics as well as clustering of risk factors were not assessed. This study aimed to determine the distribution and clustering of modifiable NCD risk factors among adults in Bhutan and their demographic and social determinants.

**Methods:**

This was secondary analysis of data from NCD Risk Factors WHO STEPS Survey 2014 in Bhutan. A weighted analysis was conducted to calculate the prevalence of NCD risk factors, and associations were explored using weighted log-binomial regression models.

**Results:**

This study included 2822 Bhutanese aged 18–69 years; 52% were 18–39 years, 62% were female, and 69% were rural resident. Prevalence of high salt intake, unhealthy diet and tobacco use were 99, 67 and 25% respectively. Raised blood pressure was the commonest (36%) modifiable biological risk factor followed by overweight (33%). The median NCD risk factors per person was 3 (Inter Quartile Range: 2–4); 52.5%% had > = 3 risk factors. A statistically significant difference was found between male vs. female in alcohol consumption(aPR 0.71, 95% CI: 0.53–0.97), low physical activity(aPR 2.06, 95% CI: 1.54–2.75), impaired fasting glycaemia(aPR 1.24, 95% CI: 1.01–1.52), and being overweight(aPR 1.46, 95% CI: 1.31–1.63). Low physical activity was more common among those with secondary and above education level vs. those without any formal education(aPR 1.71, 95% CI: 1.24–2.35), and among those residing in urban areas vs. those in rural(aPR 3.43, 95% CI: 2.27–5.18). Older participants and urban residents were more likely to have > = 3 NCD risk factors compared to younger(aPR 1.46, 95% CI: 1.35–1.58) and rural residents(aPR 1.21, 95% CI: 1.10–1.32).

**Conclusion:**

Lifestyle modifications at the population level are urgently required in Bhutan as several NCD risk factors such as high salt intake, unhealthy diet, overweight, and high blood pressure were alarmingly high and frequently clustered. Moreover there is a need to consider policy and socio-political and economic factors that have undermined global and national progress to address the rise of NCDs and their risk factors in Bhutan as elsewhere.

## Background

Non-communicable diseases (NCDs) kill 38 million people each year, and 75% of these deaths occurs in low- and middle-income countries [1]. Among the NCD-related causes of deaths almost half are due to cardiovascular diseases, a quarter to cancers, one-fifth due to diabetes, and one-tenth to respiratory diseases [1]. By 2020, it is estimated that 80% of the global burden of the diseases will be due NCDs, causing seven in ten deaths [2].

Behavioral risk factors such as tobacco use, unhealthy diet, physical inactivity, high salt consumption, and harmful use of alcohol lead to biological risk factors such as obesity, hypertension, hyperlipidemia, and impaired fasting glycaemia, which ultimately lead to NCDs. Clustering of risk factors in the same individual or population is common and this further increases the risk of NCDs [3].

The World Health Organization (WHO) in 2014 reported that 56% of the deaths in Bhutan were caused by NCD [4]. The World Bank reported that Bhutan by 2025 will have 7.3% of people above 65 years of age, which will increase the burden of NCD, since the prevalence of NCDs increases with age [5]. Bhutan is currently facing a double burden of NCDs and communicable diseases, and the administrative data collected by the Ministry of Health indicates rising trends of NCDs [6]. In line with this and the global action plan on NCD of achieving a 25% reduction in premature mortality from non-communicable diseases (NCDs) by 2025 (the 25 × 25 target) [7], Bhutan has developed the National Action Plan on NCD [8], and has rolled-out WHO Package of Essential NCDs (PEN) in the country [6]. However, the country requires robust data for planning and allocation of resources. In view of this, the Ministry of Health undertook a nationwide survey in 2014 using the WHO STEP-wise approach [9]. The aim of the survey was to determine the prevalence of key behavioral and biological risk factors of NCDs in the adult population (aged 18–69 years) of Bhutan.

The survey report did not indicate how the NCD risk factors are associated with different socio-demographic characteristics, and whether is there any pattern in clustering of risk factors [10]. Therefore, this study aimed to determine the distribution and clustering of modifiable non-communicable disease (NCD) risk factors among adults in Bhutan and their demographic and social determinants.

## Methods

### Study design

This was a secondary analysis of nation-wide cross-sectional survey data collected for NCD Risk Factors Surveillance STEPS Survey 2014 in Bhutan.

### General setting

Bhutan with an estimated population of 757,042 in 2015 [11] and an area of 38,394 km^2^ [12] is located between India to the south and China to the north. It is divided into 20 districts, which include Thimphu, the capital city located at the western side of the country. Bhutan measures Gross National Happiness (GNH) Index for a holistic approach towards sustainable development by giving equal importance to non-economic aspects of wellbeing in addition to economic aspects [13]. This aspect of wellbeing in the GNH concept has given huge importance to health in Bhutan, that the country provides free access to basic public health services in both modern and traditional medicines [14].

### Non-communicable disease division

NCD Division is a part of Department of Public Health under the Ministry of Health that looks after various NCD prevention programs such as adolescent health, disability prevention, life-style related disease, mental health, nutrition, reproductive health, village health worker and suicide prevention. The life-style related disease program, in collaboration with WHO conducted the national survey for noncommunicable disease risk factors and mental health using WHO STEPS approach in Bhutan, [10]. The survey has provided information on nine modifiable NCD risk factors, and has helped program to design and come up with policy interventions.

### Study population

Adult Bhutanese men and women aged 18–69 years in 2014.

### Data variables

The variable used from the survey were nine modifiable behavioral (tobacco use, alcohol consumption, low consumption of fruits and/or vegetables, low physical activity, and high salt intake) and biological (overweight, raised blood pressure, raised cholesterol level and impaired fasting glycaemia) NCD risk factors (refer Table 1a and b for the definitions of these variables). The socio-demographic characteristics variables were age, sex, education, employment, annual income tertiles, and urban–rural stratification. The nine modifiable NCD risk factors were used to create a new variable based on how it clustered. Co-existence of any three or more (≥3) NCD behavioral or biological risk factors in the same individual was defined as clustering of NCD risk factors for this study.

**Table 1:** Definition used for the modifiable NCD risk factors for this study

a Modifiable Behavioral NCD Risk Factor		
S.no	NCD risk factor	Definition
1	Tobacco use	Smoker: Currently smokes any tobacco products, such as cigarettes, cigars, or pipes, etc. Smokeless: Currently use any smokeless tobacco such as snuff, chewing tobacco, betel, etc. A detailed study using NCD STEPS data for tobacco use has already been done and it is currently under review for publication. Therefore this study will not focus on tobacco use; any information required will be used from that study. However, this variable will be used during the analysis of clustering of NCD risk factors.
2	High alcohol consumption	Current alcohol drinker (intermediate or high) Those who are current alcohol drinker and drinks pure alcohol above 40 g for men and above 20 g for women on average per occasion.
3	Low consumption of fruit and vegetable	Less than five serving of fruit and/or vegetable on an average day In a typical week, number of days the respondent ate fruit and/or vegetable, and number of servings of vegetables they ate on one of those days.
4	Low physical activity	Percentage of respondents not meeting WHO recommendations on physical activity for health (respondents doing less than 150 min of moderate-intensity physical activity per week, or equivalent).
5	Salt intake	Levels of sodium and creatinine in spot urine samples are used to estimate population 24 h salt intake, using the INTERSALT equation: Males: 23.51 + 0.45*spot Na concentration (mmol/L) -3.09*spot creatinine concentration (mmol/L) + 4.16*BMI + 0.22*Age Females: 3.74 + 0.33* spot Na concentration (mmol/L)-2.44* spot creatinine concentration (mmol/L) + 2.42* BMI +2.34* Age − 0.03* Age ^2 The 24 h sodium values in mmol are divided by 17.1 in order to get grams of salt.
b Modifiable Biological NCD Risk Factor		
1	Overweight	Overweight is defined as Body Mass Index ≥25 kg/m^2^
2	Raised blood pressure	Raised blood pressure individuals are those with SBP ≥140 and/or DBP ≥ 90 mmHg or currently on medication for raised blood pressure. This study took BP 3 measurements and for the purpose of this study, the average reading of these three measurements will be used.
3	Impaired fasting glycaemia	Impaired fasting glycaemia is defined as those with capillary whole blood value: ≥5.6 mmol/L (100 mg/dl) and <6.1 mmol/L (110 mg/dl)
4	Raised fasting cholesterol level	Raised fasting cholesterol level is defined as total cholesterol level ≥ 5.0 mmol/L or ≥190 mg/dl or currently on medication for raised cholesterol

### Sampling and sample size

Data collected during STEPS survey 2014 using pilot-tested and validated structured questionnaire (WHO STEP-wise method) between April to June 2014 was used for this study. The survey used a multi-stage sampling method to recruit participants, and the targeted sample size was 2912. The response rate of the survey was 97%. More information on sampling and sample size calculation is available in the STEPS survey report, 2014 [10].

### Analysis and statistics

The categorical variables were described using numbers and proportions, and continuous variables using mean and standard deviation, or median and interquartile range where applicable. A weighted analysis [15] was conducted to calculate the prevalence of NCD risk factors. Equiplots [16] were utilized to show the disparity among socio-demographic characteristics and the NCD risk factors. Associations between NCD modifiable risk factors and selected socio-demographic characteristics of the participants were explored using weighted log-binomial regression models. *P*-value of <0.05 was considered statistically significant. Clustering of NCD risk factors was assessed and the occurrence of three risk factors or more was used as an outcome variable to fit a weighted log-binomial regression model to explore associations between clustering and selected socio-demographic characteristics. The variance inflation factor (VIF) values for each predictor were calculated as a check for multicollinearity. The sensitivity analysis was also conducted by removing those already diagnosed with hypertension and on medication, and those already diagnosed with diabetes and on medication, or those already diagnosed with high cholesterol and on medications from the models, but it did not show any significant difference in the estimates. Therefore, for the final model they are included in the analysis. The analysis was conducted using STATA version 12 (Statacorp, College Station, TX, USA).

## Results

A total of 2822 adults participated in the survey, 97% of 2912 targeted sample size. Among them, 62% were female, 69% were residing in rural areas, 63% had no formal education, 57% were from the poorest income level, and 55% were self-employed. The detailed socio-demographic characteristics are described in Table 2.

**Table 2:** Socio-demographic characteristics of the non-communicable disease risk factor survey participants, Bhutan 2014

Characteristics	Number (n)	Percentage (%)
Total	2822	100%
Age group (years)		
18–39	1469	52
40–65	1353	48
Gender		
Male	1074	38
Female	1748	62
Education Level		
None	1766	63
Primary	632	22
Secondary and above	421	15
Employment		
Salaried (Non/Government employee)	480	17
Self employed	1558	55
Unpaid	782	28
Area of Residence		
Rural	1952	69
Urban	870	31
Income Level (in USD)^a^		
Poorest (<=1147)	1562	57
Middle (1148–2823)	758	28
Richest (> = 2824)	419	15

^a^tertiles of annual income reported by the household, exchange rate @Nu. 68 per 1 USD

Table 3 presents the prevalence of five modifiable behavioral risk factors. Ninety-nine per cent of the survey participants had high salt intake; the mean salt consumption was 9 g per person per day (standard deviation, 2.01). Unhealthy diet and tobacco use were reported by 67 and 25% respectively. A fraction of 7% of adult Bhutanese consumes above 40 g (for men) and above 20 g (for women) of pure alcohol per occasion. The prevalence of behavioral risk factors did not vary vastly between the different socio-demographic sub-groups, except for low physical activity: 10% of females reported less than 150 min of moderate-intensity physical activity per week, or equivalent, compared to 4% of males. Tobacco use, varied significantly only between males and females (34% vs. 14%, *p* < 0.05) [17].

**Table 3:** Prevalence^a^ of modifiable behavioral non-communicable disease risk factors stratified by socio-demographic characteristics in Bhutan, 2014

Socio-demographic characteristics	High alcohol consumption	High salt intake	Unhealthy diet	Low physical activity
	m/n(%)	m/n(%)	m/n(%)	m/n(%)
National	189/2756 (7)	2592/2618 (99)	1901/2817 (67)	207/2742(6)
Age group (years)				
18–39	71/1444 (6)	1318/1329 (99)	988/1469 (67)	116/1437 (6)
40–65	118/1312 (9)	1274/1289 (99)	913/1348 (67)	91/1305 (7)
Gender				
Male	98/1046 (8)	1015/1018 (99)	702/1071 (65)	54/1033 (4)
Female	91/1710 (5)	1577/1600 (99)	1199/1746 (70)	153/1709 (10)
Education Level				
None	145/1716 (8)	1633/1655 (99)	1195/1763 (68)	105/1709 (5)
Primary	33/623 (6)	583/586 (99)	431/632 (67)	44/620 (5)
Secondary and above	10/416 (5)	375/376 (100)	274/421 (65)	58/412 (13)
Employment				
Salaried (Non/Govt. employee)	27/489 (7)	453/456 (99)	346/496 (66)	44/486 (7)
Self employed	135/1508 (8)	1450/1467 (99)	956/1557 (63)	83/1497 (4)
Unpaid	27/759 (4)	689/695 (99)	599/764 (77)	80/759 (10)
Area of Residence				
Rural	164/1894 (8)	1801/1820 (99)	1342/1949 (67)	79/1886 (3)
Urban	25/862 (5)	791/798 (99)	559/868 (68)	128/856 (13)
Income Level (in USD)^b^				
Poorest (<=1147)	133/1517 (8)	1436/1454 (99)	1050/1560 (68)	72/1511 (4)
Middle (1148–2823)	38/747 (6)	694/700 (99)	522/757 (64)	70/743 (8)
Richest (> = 2824)	13/415 (4)	381/383 (99)	265/419 (66)	60/411 (13)

*Tobacco user: Refer study (Current tobacco use and its associated factors among adults in a country with comprehensive ban on tobacco: findings from the nationally representative STEPS survey, Bhutan, 2014

^a^Prevalence rates are weighted

^b^Tertiles of annual income earned by the household; exchange rate @Nu. 68 per 1 USD

**Fig. 1:**
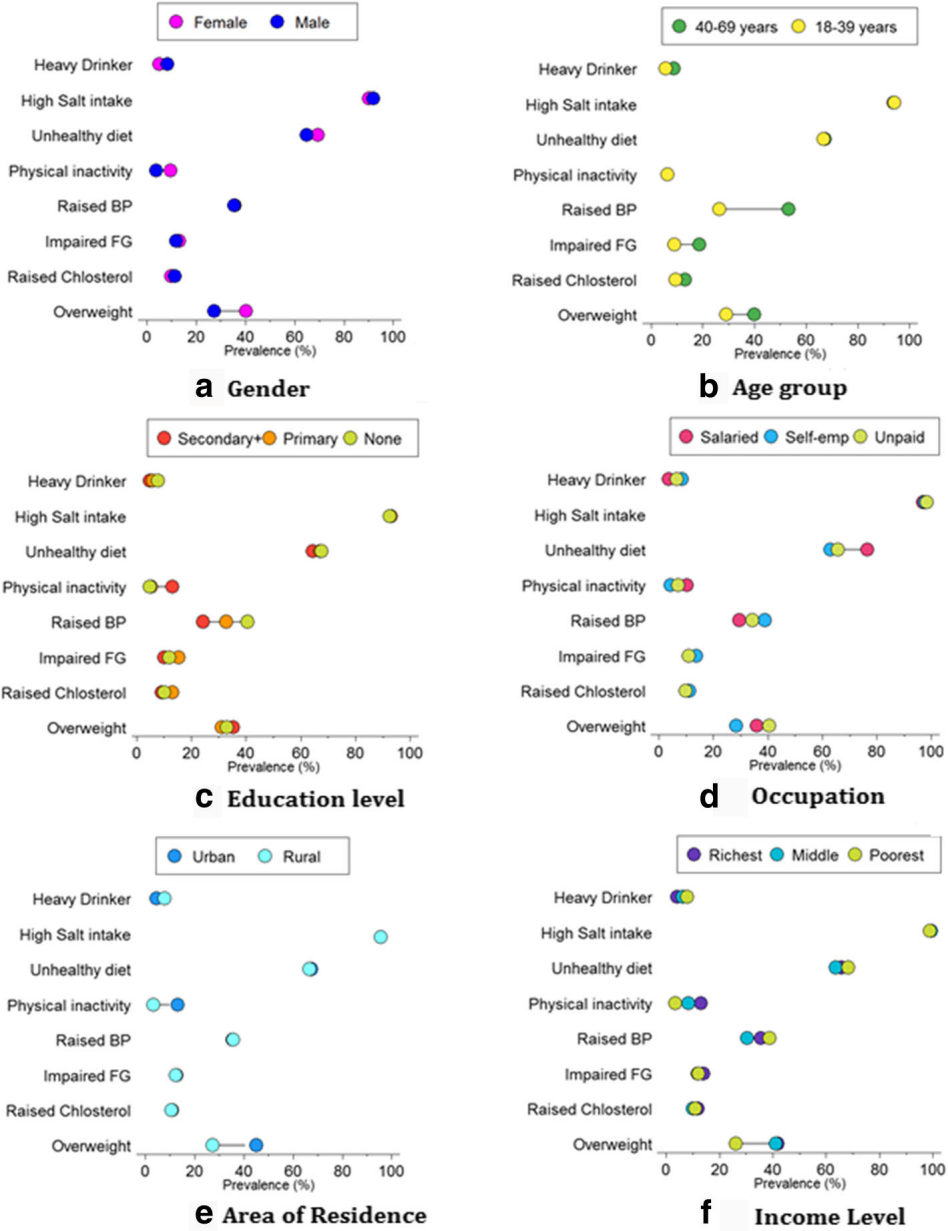
Equiplots showing disparity of noncommunicable disease modifiable risk factors among different socio-demographic characteristics. **a** Gender **b** Agegroup **c** Education Level **d** Occupation **e** Rural and Urban **f** Income Level

Among the four modifiable biological noncommunicable disease risk factors, shown in Table 4, raised blood pressure was the commonest (36%) followed by overweight (33%). Prevalence of all four risk factors was higher in the older age-group (40–65 years). The prevalence of being overweight is higher in females (40%), those residing in urban areas (45%), and those in middle & richest income level. Figure 1 shows the differences of each of the modifiable risk factors stratified by the different socio-demographic characteristics.

**Table 4:** Prevalence^a^ of modifiable biological non communicable disease risk factors stratified by socio-demographic characteristics in Bhutan, 2014

Socio-demographic characteristics	Raised Blood Pressure	Raised Impaired Fasting Glycaemia	Raised Cholesterol Level	Overweight
	m/n(%)	m/n(%)	m/n(%)	m/n(%)
National	1101/2816 (36)	287/2724 (11)	374/2761 (13)	1050/2739 (33)
Age group(years)				
18–39	381/1467 (26)	128/1419 (9)	132/1434 (10)	493/1400 (29)
40–65	720/1349 (53)	159/1305 (19)	242/1327 (13)	557/1339 (40)
Gender				
Male	423/1071 (36)	109/1037 (12)	135/1049 (11)	320/1067 (27)
Female	678/1745 (36)	178/1687 (13)	239/1712 (10)	730/1672 (40)
Education Level				
None	783/1764 (41)	174/1712 (12)	230/1734 (10)	660/1724 (33)
Primary	215/632 (33)	75/608 (15)	101/619 (13)	241/612 (31)
Secondary and above	102/419 (24)	37/401 (10)	43/405 (9)	148/402 (35)
Employment				
Salaried (Non/Govt. employee)	189/494 (34)	49/473 (11)	57/482 (10)	192/484 (41)
Self employed	661/1557 (39)	156/1526 (14)	224/1532 (11)	554/1522 (28)
Unpaid	251/765 (30)	81/723 (11)	93/745 (11)	304/733 (36)
Area of Residence				
Rural	778/1950 (36)	192/1887 (12)	265/1915 (11)	629/1905 (28)
Urban	323/866 (35)	95/837 (13)	109/846 (11)	421/834 (45)
Income Level (in USD)^b^				
Poorest (<=1147)	650/1561 (39)	155/1509 (12)	208/1534 (11)	494/1518 (26)
Middle (1148–2823)	262/757 (30)	72/735 (12)	89/745 (10)	336/731 (41)
Richest(> = 2824)	154/417 (36)	50/398 (14)	59/399 (12)	195/409 (42)

^a^Prevalence rates are weighted

^b^Tertiles of annual income earned by the household; exchange rate @Nu. 68 per 1 USD

Tables 5 and 6, show the adjusted and unadjusted prevalence ratios of behavioral and biological risk factors respectively. A statistically significant difference was found between male vs. female in alcohol consumption (aPR 0.71, 95% CI: 0.53–0.97), low physical activity (aPR 2.06, 95% CI: 1.54–2.75), impaired fasting glycaemia (aPR 1.24, 95% CI: 1.01–1.52), and being overweight (aPR 1.46, 95% CI: 1.31–1.63). Low physical activity was more common among those with secondary and above education level compared to those without any formal education (aPR 1.71, 95% CI (1.24–2.35), and among those residing in urban areas vs. those in rural (aPR 3.43, 95% CI 2.27–5.18). Similarly, the prevalence of raised blood pressure was higher among the urban vs. rural (aPR 1.2, 95% CI: 1.06–1.36); and being overweight was more common among urban vs. rural residents (aPR 1.36, 95% CI 1.21–1.54), and among middle (aPR 1.47, 95% CI 1.28–1.68) and richest (aPR 1.47, 95% CI 1.26–1.72) income level vs. poorest income level.

**Table 5:** Adjusted and unadjusted prevalence-ratio of behavioral noncommunicable disease risk factors and the socio-demographic characteristics, Bhutan 2014

Socio-demographic characteristics	High alcohol consumption		Unhealthy diet		Low physical activity	
	PR (95%CI)	aPR (95%CI)	PR (95%CI)	aPR (95%CI)	PR (95%CI)	aPR (95%CI)
Age group						
18–39	1	1	1	1	1	1
40–65	1.53(1.48, 1.58)^**^	1.26(0.97, 1.65)	0.98(0.97, 1)^*^	0.98(0.87, 1.1)	1.02(0.99, 1.06)	1.42(1.1, 1.85)^*^
Gender						
Male	1	1	1	1	1	1
Female	0.6(0.58, 0.62)^**^	0.71(0.53, 0.97)^*^	0.86(0.85, 0.87)^**^	0.94(0.84, 1.06)	2.46(2.37, 2.54)^**^	2.06(1.54, 2.75)^**^
Education Level						
None	1	1	1	1	1	1
Primary	0.76(0.73, 0.79)^**^	0.79(0.55, 1.13)	1.01(1, 1.03)	0.95(0.83, 1.08)	1.09(1.04, 1.14)^**^	0.93(0.66, 1.31)
Secondary and above	0.61(0.58, 0.64)^**^	0.96(0.6, 1.53)	1.09(1.08, 1.11)^**^	1.06(0.89, 1.26)	2.66(2.56, 2.76)^**^	1.71(1.24, 2.35)^**^
Employment						
Salaried (Non/Govt employee)	1	1	1	1	1	1
Self employed	1.27(1.22, 1.32)^**^	1.06(0.72, 1.56)	1.08(1.06, 1.09)^**^	1.19(1.02, 1.39)^*^	0.61(0.58, 0.64)^**^	1.48(1.05, 2.08)^*^
Unpaid	0.54(0.51, 0.57)^**^	0.55(0.3, 1.03)	0.69(0.67, 0.7)^**^	0.77(0.63, 0.94)^*^	1.44(1.39, 1.5)^**^	1.73(1.27, 2.34)^**^
Area of Residence						
Rural	1	1	1	1	1	1
Urban	0.58(0.56, 0.61)^**^	0.76(0.51, 1.13)	0.97(0.96, 0.98)^**^	0.94(0.82, 1.09)	4.04(3.9, 4.18)^**^	3.43(2.27, 5.18)^**^
Income Level (in USD)^b^						
Poorest (<=1147)	1	1	1	1	1	1
Middle (1148–2823)	0.82(0.79, 0.85)^**^	0.92(0.67, 1.27)	1.15(1.14, 1.17)^**^	1.22(1.07, 1.4)^**^	2.37(2.27, 2.46)^**^	1.01(0.73, 1.38)
Richest (> = 2824)	0.54(0.51, 0.57)^**^	0.64(0.36, 1.15)	1.09(1.07, 1.11)^**^	1.1(0.92, 1.31)	3.71(3.55, 3.87)^**^	1.19(0.87, 1.64)

*PR* prevalence ratio, *aPR* adjusted prevalence ratio

^*^Statistically significant at <0.05, ^**^ statistically significant at <0.001

^b^tertiles of annual income reported by the household; exchange rate @Nu. 68 per 1 USD

**Table 6:** Adjusted and unadjusted prevalence-ratio of biological noncommunicable disease risk factors and the socio-demographic characteristics, Bhutan 2014

Socio-demographic characteristics	Raised Blood Pressure		Impaired Fasting Glycaemia		Raised Cholesterol		Overweight	
	PR (95%CI)	aPR (95%CI)	PR (95%CI)	aPR (95%CI)	PR (95%CI)	aPR (95%CI)	PR (95%CI)	aPR (95%CI)
Age group								
18–39	1	1	1	1	1	1	1	1
40–65	2.02(2, 2.04)^**^	1.89(1.69, 2.11)^**^	2.06(2.01, 2.11)^**^	2.18(1.77, 2.69)^**^	1.49(1.46, 1.53)^**^	1.64(1.3, 2.08)^**^	1.38(1.36, 1.39)^**^	1.55(1.39, 1.72)^**^
Gender								
Male	1	1	1	1	1	1	1	1
Female	1.01(1, 1.02)	1.08(0.98, 1.19)	1.12(1.09, 1.15)^**^	1.24(1.01, 1.52)^*^	0.88(0.85, 0.9)^**^	0.85(0.67, 1.08)	1.48(1.46, 1.5)^**^	1.46(1.31, 1.63)^**^
Education Level								
None	1	1	1	1	1	1	1	1
Primary	0.81(0.8, 0.82)^**^	0.92(0.82, 1.04)	1.28(1.25, 1.31)^**^	1.65(1.33, 2.05)^**^	1.28(1.24, 1.31)^**^	1.49(1.17, 1.91)^**^	0.95(0.93, 0.96)^**^	0.97(0.86, 1.1)
Secondary and above	0.6(0.59, 0.61)^**^	0.78(0.64, 0.95)^*^	0.86(0.83, 0.89)^**^	1.13(0.76, 1.68)	0.87(0.84, 0.9)^**^	1.01(0.67, 1.53)	1.07(1.05, 1.09)^**^	0.88(0.75, 1.04)
Employment								
Salaried (Non/Govt employee)	1	1	1	1	1	1	1	1
Self employed	1.13(1.12, 1.15)^**^	0.92(0.79, 1.06)	1.25(1.22, 1.29)^**^	1.47(1.07, 2)^*^	1.11(1.07, 1.14)^**^	1.11(0.8, 1.55)	0.7(0.69, 0.71)^**^	0.84(0.73, 0.96)^*^
Unpaid	0.86(0.84, 0.87)^**^	0.82(0.7, 0.97)^*^	1(0.97, 1.04)	1.19(0.83, 1.69)	1.12(1.08, 1.16)^**^	1.26(0.89, 1.78)	0.89(0.87, 0.9)^**^	0.86(0.74, 0.99)^*^
Area of Residence								
Rural	1	1	1	1	1	1	1	1
Urban	0.99(0.98, 1)^*^	1.2(1.06, 1.36)^**^	1.03(1, 1.05)^*^	1.15(0.91, 1.46)	1.07(1.04, 1.1)^**^	1.18(0.91, 1.53)	1.64(1.62, 1.66)^**^	1.36(1.21, 1.54)^**^
Income Level (in USD)^a^								
Poorest (<=1147)	1	1	1	1	1	1	1	1
Middle (1148–2823)	0.78(0.77, 0.79)^**^	0.85(0.75, 0.97)^*^	0.98(0.95, 1)	1.12(0.88, 1.44)	0.93(0.9, 0.96)^**^	0.98(0.73, 1.31)	1.58(1.56, 1.6)^**^	1.47(1.28, 1.68)^**^
Richest(> = 2824)	0.92(0.9, 0.93)^**^	1.02(0.88, 1.19)	1.16(1.12, 1.2)^**^	1.15(0.86, 1.54)	1.13(1.09, 1.17)^**^	1.14(0.83, 1.57)	1.61(1.58, 1.63)^**^	1.47(1.26, 1.72)^**^

*PR* prevalence ratio, *aPR* adjusted prevalence ratio

*Statistically significant at <0.05, **statistically significant at <0.001

^a^tertiles of annual income reported by the household; exchange rate @Nu. 68 per 1 USD

**Fig. 2:**
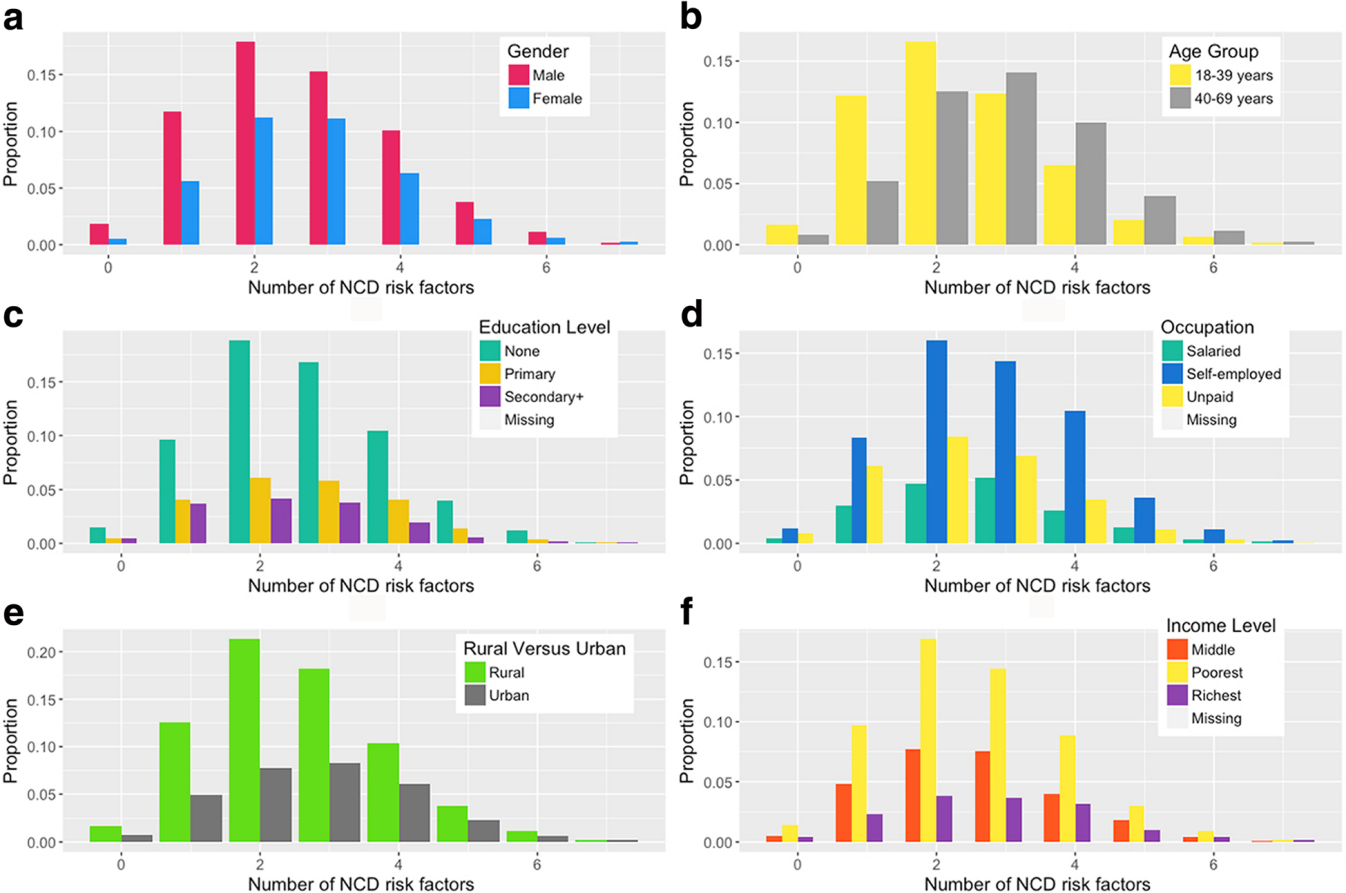
Clustering of noncommunicable disease modifiable risk factors stratified by different socio-demographic characteristics. **a** Gender **b** Agegroup **c** Education Level **d** Occupation **e** Rural and Urban **f** Income Level

Figure 2 presents how number of risk factors clusters among different socio-demographic characteristics. The association of socio-demographic characteristics with clustering of 3 or more risk factors is shown in Table 7. Older participants were 46% more likely to have 3 or more risk factor compared to younger (aPR 1.46, 95% CI: 1.35–1.58), while urban residents were 21% more likely to have 3 or more risk factor compared to rural residents (aPR 1.21, 95% CI: 1.10–1.32).

**Table 7:** Adjusted and unadjusted prevalence-ratios of 3 or more cluster of NCD risk factors and the socio-demographic characteristics, Bhutan 2014

Socio-demographic Characteristics	3 or more risk factor	
	PR (95%CI)	aPR (95%CI)
Agegroup		
18–39	1	1
40–65	1.44 (1.43, 1.45)^**^	1.46 (1.35, 1.58)^**^
Gender		
Male	1	1
Female	0.93 (0.92, 0.94)^**^	0.98 (0.90, 1.06)
Education Level		
None	1	1
Primary	1.03 (1.02, 1.04)^**^	1.06 (0.97, 1.16)
Secondary and above	0.91 (0.90, 0.92)^**^	0.94 (0.82, 1.07)
Employment		
Salaried (Non/Govt. employee)	1	1
Self employed	0.90 (0.89, 0.91)^**^	0.98 (0.89, 1.09)
Unpaid	0.77 (0.76, 0.78)^**^	0.87 (0.77, 0.97)^*^
Area of Residence		
Rural	1	1
Urban	1.19 (1.18, 1.20)^**^	1.21 (1.10, 1.32)^**^
Income Level (in USD)^a^		
Poorest (<=1147)	1	1
Middle (1148–2823)	1.14 (1.13, 1.15)^**^	1.12 (1.02, 1.23)^*^
Richest (> = 2824)	1.13 (1.12, 1.14)^**^	1.14 (1.01, 1.28)^*^

*PR* prevalence ratio, *aPR* adjusted prevalence ratio

^*^Statistically significant at <0.05, ^**^ statistically significant with <0.001

^a^tertiles of annual income reported by the household; exchange rate @Nu. 68 per 1 USD

## Discussion

This secondary analysis of the first nation-wide survey data on NCD risk factors in Bhutan is the first to describe the distribution and clustering of modifiable behavioral and biological risk factors. Not only was the prevalence of most risk factors found to be high in this population, but also clustering of risk factors was widely prevalent. Almost every adult Bhutanese consumes high amount of salt. Two out of three adults has an unhealthy diet, one in three is overweight and one in three has raised blood pressure. Despite a national ban on tobacco since 2004, one in three adult men does smoke or uses smokeless tobacco, and this has been already described [17]. The median number of risk factor per adult was three, while the most commonly clustered factors was a combination of high salt intake, unhealthy diet, overweight and high blood pressure.

The mean amount of salt consumed is 9 g per day, which is almost double the amount of what WHO recommends (<5 g per day) [18]. Bhutan however is not much different in terms of high salt intake compared to other countries. A study conducted in Shandong province of China found high salt intake among 92% of its adult population [19], while most countries in South East Asia Region consume more than 10 g/day of salt – Sri Lanka 9–11 g/day; Nepal 10–13 g/day; Indonesia 15 g/day; Bangladesh 17 g/day [20].

A number of studies have indicated that salt intake is highly associated with development of hypertension –one of the NCD biological risk factors [21–24], this may partly explain the high prevalence of raised blood pressure as indicated by this study (36%). Therefore, one of the key areas of intervention for addressing the growing burden of NCD in Bhutan should be reduction of salt intake. The successful salt intake endeavors could save millions of dollar besides bringing enormous public health benefits. It has been calculated that reducing sodium intake to 2.3 g/day could save US$ 18 billion in annual US healthcare costs [25], and gold standard national sodium reduction program would cost 1–2% of hypertension program cost [20]; however population-based interventions apart from awareness campaigns are not commonly reported. A small-scale pilot study in South Korea has shown that educating restaurant owners and cooks to lower their own sodium intake to be a potential strategy for reducing the sodium contents of restaurant food [26]. However, more evidence is required to see whether this particular strategy would work in Bhutan as this survey reports that, respondents consumed on average only one meal per week that was not prepared at home [10].

Bhutan, being a mountainous country nestled in the Eastern Himalaya availability of fruits and vegetables are seasonal. Many respondents said that they eat plenty of fruits when it is available at their farm or locality and they don’t eat any fruit when it is not available. Given the harsh climatic conditions hardly anything grows in winter in most part of the country. People either depend on the imported fruits and vegetables which are often not accessible to many or they resort to traditional cuisine of potatoes and dried vegetables. Moreover, because of the self-imposed isolation from modern civilization up until 1960s Bhutanese had very little exposure to the latest evidence on healthy diet. Fruits were considered as a snack to be munched on if you find one, but not as a nutritious food. As a result, consuming the recommended five servings of fruits and vegetables on an average day was found to be uncommon among adult Bhutanese. Other WHO STEPS surveys from the region have found similar results, however in high-income countries the case has been different [27].

We found low physical activity (i.e. not meeting WHO recommended ≥150 min of moderate-intensity activity per week, or equivalent) to be low at 6% among the Bhutanese population, which is three time less than the global estimate for prevalence of physical inactivity among adults 23% [24]. This finding might be because 70% of respondents in the survey were from rural areas, where people have to do hard laborious work in the fields. The 2007 STEPS survey conducted only in the urban area of Thimphu (capital city) has found that approximately 60% were not attaining the minimum required physical activity. Those with education level above secondary have higher prevalence of low physical activity; this might be probably because those with higher education level live in urban areas. Numerous studies have found that the combination of unhealthy diet and low physical activity are associated with overweight and obesity, which ultimately are risk factors for diabetes and cardiovascular disease.

The clustering of 3 or more risk factors is higher among adults older than 40 years old and among urban residents. Since one risk factor seems to lead to another, it is important to tackle NCD risk factors at behavioral level. There have been reports from urban Indian, Brazilian, and North American populations on clustering of risk factors, especially cardiovascular factors among hypertensive patients [3, 28, 29]. A Bangladeshi secondary analysis of a national STEPS survey has found that that 38% of the population had at least three risk factors and clustering was associated with age, male sex, urban residence, quality of house and educational level. The clustering phenomenon may predispose to a higher burden of NCDs compared to populations with lower tendency of clustering, Nevertheless, this represents not only a public health challenge but also an opportunity; interventions targeting more than one risk factors and tailored to the needs of specific subgroups and populations may be combined and resources may be shared and used more efficiently.

This study has several strengths and limitations. First, the survey that the data were derived from was conducted at the national level and therefore the surveyed sample was representative of Bhutanese adults aged 18–69 years. Pilot tested & validated instruments were used and the survey enumerators where trained thoroughly on data collection. Supervisors were sent in the fields and spot checks were done, to minimize non-sampling errors. Second, since the response rate was very high (97%) and weighted analysis was used to adjust for the complex survey design, the findings can be extrapolated to the whole of Bhutan. Third, we have comprehensively assessed and modeled the socioeconomic factors associated with NCD risk factors and we looked at clustering patterns for the first time in the country. Lastly, we adhered to Strengthening the Reporting of Observational Studies in Epidemiology (STROBE) guidelines in conducting and reporting the study [30].

An important limitation of the study was the potential social desirability bias in self-reporting tobacco use and this was probably enhanced by the fact that the survey enumerators were health care providers and that tobacco products are banned and the use of tobacco in public is prohibited. The prevalence of smoking might have therefore been underestimated. Second, the sampling framework was based on the 2005 census when the urban to rural ratio was 30:70 but this might have changed at the time of the survey. The prevalence of high salt intake was calculated based on single spot urine sample rather than gold standard 24-h urine sample collection, which might have led to inaccurate estimation [31]. Lastly, our study has the inherited limitations of the cross-sectional design.

There are some recommendations that could be made based on the evidence produced by this study. First, this study highlighted the most prevalent modifiable NCD risk factors, their determinants and their clustering patterns and therefore it may inform the national NCD Division in priority setting and allocation of recourses. Second, as several of the prevalent risk factors are behavioral and tend to cluster, the public and high-risk sub-groups and populations should be educated through culturally appropriate and innovative public health messaging and mass media campaigns. Targeted interventions in educational institutions and places frequented by the youth should be initiated and strengthened in order to influence long terms positive life-style changes. Third, health advice and care should be given to patients of any NCDs or NCD factors during any contact with health care providers. Teachers and health care providers could be trained in counseling. Fourth, legislation and enforcement of existing legislation (such as the tobacco ban) should be strictly enforced. The Ministry of Health in collaboration with Ministry of Trade and Industry could set salt limits for the food industry in the country, as 1 in 10 adult Bhutanese consume processed food high in salt daily [10]. Finally, innovation and piloting should be encouraged and expanded; for example an initiative of the Ministry of Health (December 2016) to offer aerobics, yoga and table-tennis classes after office hours to the headquarter staff could be encouraged to expand to other ministries, sectors and corporate agencies.

## Conclusions

The prevalence of modifiable behavioral and biological NCD risk factors was high among Bhutanese adults with a strong tendency of clustering. If a risk factor is identified at the level of the individual or the population, a rigorous assessment of other risk factors has to be made at both the clinical and public health settings. Lifestyle modifications at the population level are urgently required as several risk factors such as high salt intake, unhealthy diet, obesity, and high blood pressure were alarmingly high and frequently clustered. This represents an emerging public health threat to the population, the health system as well as the economy of Bhutan. Moreover there is a need to consider policy and socio-political and economic factors that have undermined global and national progress to address the rise of NCDs and their risk factors in Bhutan as elsewhere.

## Abbreviations

aPR: Adjusted prevalence ratio
NCD: Noncommunicable disease
PEN: Package of essential NCDs
PR: Prevalence ratio
WHO: World Health Organization

## References

[1] World Health Organization. Noncommunicable diseases factsheet http://www.who.int/mediacentre/factsheets/fs355/en (2015). Accessed 3 Mar 2016.

[2] Boutayeb A, Boutayeb S (2005). The burden of non communicable diseases in developing countries. Int J Equity Health.

[3] Zaman MM, Bhuiyan MR, Karim MN, MoniruzZaman RMM, Akanda AW (2015). Clustering of non-communicable diseases risk factors in Bangladeshi adults: an analysis of STEPS survey 2013. BMC Public Health.

[4] World Health Organization. Noncommunicable Diseases (NCD) Country Profiles. 2014. http://www.who.int/nmh/countries/btn_en.pdf?ua=1. Accessed 3 Mar 2016.

[5] The World Bank. NCDs Policy Brief - Bhutan. http://siteresources.worldbank.org/SOUTHASIAEXT/Resources/223546-1296680097256/7707437-1296680114157/NCD_BH_Policy_Feb_2011.pdf (2011). Accessed 3 Mar 2016.

[6] Royal Government of Bhutan Ministry of Health. Annual health bulletin 2014. http://www.health.gov.bt/wp-content/uploads/ftps/annual-health-bulletins/ahb2014/ahbContent2014.pdf. 27th ed; (2015). Accessed 3 Mar 2016.

[7] Kontis V, Mathers CD, Bonita R, Stevens GA, Rehm J, Shield KD (2015). Regional contributions of six preventable risk factors to achieving the 25 X 25 non-communicable disease mortality reduction target: a modelling study. Lancet Glob. Heal..

[8] Royal Government of Bhutan Ministry of Health. The Multisectoral National Action Plan for the Prevention and Control of Noncommunicable diseases 2015–2020. http://www.health.gov.bt/wp-content/uploads/moh-files/2015/12/The-Multisectoral-National-Action-Plan-for-the-Prevention-and-Control-of-NCDs-2015-2020.pdf. Accessed 12 Dec 2016.

[9] World Health Organization. STEPwise approach to surveillance (STEPS). http://www.who.int/chp/steps/en (2015). Accessed 2016 Dec 12.

[10] World Health Organization. STEPwise approach to surveillance (STEPS). http://www.who.int/chp/steps/Bhutan_2014_STEPS_Report.pdf (2014). Accessed 12 Dec 2016.

[11] National Statistical Bureau of Bhutan. Dzongkhag population projections 2006–2015 Thimphu, Bhutan. http://www.nsb.gov.bt/publication/files/pub3uu3600pb.pdf (2008). Accessed 4 Mar 2016.

[12] World Bank. Surface area. http://data.worldbank.org/indicator/AG.SRF.TOTL.K2. Accessed 4 Mar 2016.

[13] The Centre for Bhutan Studies & GNH Research. The 2010 Bhutan gross National Happiness Index. http://www.grossnationalhappiness.com/articles (2011). Accessed 3 Mar 2016.

[14] Tobgay T, Dophu U, Torres CE, Na-Bangchang K (2011). Health and gross National Happiness: review of current status in Bhutan. J Multidiscip Healthc.

[15] Bethlehem J. Applied survey methods - a statistical perspective. http://www.applied-survey-methods.com/weight.html. Accessed 8 Sep 2017.

[16] Equiplot - Int’l Center for Equity in Health. http://www.equidade.org/equiplot. Accessed 12 Dec 2016.

[17] Gurung MS, Pelzom D, Dorji T, Drukpa W, Wangdi C. Current tobacco use and its associated factors among adults in a country with comprehensive ban on tobacco : findings from the nationally representative STEPS survey, Bhutan, 2014. Popul Health Metrics. 2016:1–9. 10.1186/s12963-016-0098-9 PMC4977656 27507928

[18] World Health Organization. Guideline sodium intake for adults and children sodium intake for adults and children. http://apps.who.int/iris/bitstream/10665/77985/1/9789241504836_eng.pdf?ua=1&ua=1. Accessed 14 Dec 2016.

[19] Xu J, Wang M, Chen Y, Zhen B, Li J, Luan W (2014). Estimation of salt intake by 24-hour urinary sodium excretion: a cross-sectional study in Yantai, China. BMC Public Health.

[20] SEARO W. Expert meeting on population sodium reduction strategies for prevention and control of NCDs in South East Asia region. http://www.searo.who.int/entity/noncommunicable_diseases/documents/sea_ncd_88.pdf (2012). Accessed 14 Dec 2016.

[21] Thomas MC, Moran J, Forsblom C, Harjutsalo V, Thorn L, Ahola A (2011). The association between dietary sodium intake, ESRD, and all-cause mortality in patients with type 1 diabetes. Diabetes Care.

[22] Ha SK (2014). Dietary salt intake and hypertension. ISSN Electrolyte Blood Press.

[23] World Health Organization. Sodium intake for adults and children. http://www.who.int/elena/titles/guidance_summaries/sodium_intake/en (2014). Accessed 14 Dec 2016.

[24] World Health Organization. Global status report on noncommunicable diseases 2014 http://apps.who.int/iris/bitstream/10665/148114/1/9789241564854_eng.pdf. Accessed 2 Mar 2016. 10.1161/STROKEAHA.115.008097 25873596

[25] Wang G, Labarthe D (2011). The cost-effectiveness of interventions designed to reduce sodium intake. J Hypertens.

[26] Park S, Lee H, Seo D-I, Oh K-H, Hwang TG, Choi BY (2016). Educating restaurant owners and cooks to lower their own sodium intake is a potential strategy for reducing the sodium contents of restaurant foods: a small-scale pilot study in South Korea. Nutr Res Pract.

[27] Miller V, Yusuf S, Chow CK, Dehghan M, Corsi DJ, Lock K (2016). Availability, affordability, and consumption of fruits and vegetables in 18 countries across income levels: findings from the prospective urban rural epidemiology (PURE) study. Lancet Glob Heal.

[28] Barreto SM, Passos VMA, Firmo JOA, Guerra HL, Vidigal PG, Lima-Costa MFF (2001). Hypertension and clustering of cardiovascular risk factors in a community in Southeast Brazil: the Bambuí health and ageing study. Arq Bras Cardiol.

[29] Snehalatha C, Ramachandran A, Satyavani K, Sivasankari S (2003). Clustering of cardiovascular risk factors in impaired fasting glucose and impaired glucose tolerance. Diabetes Care.

[30] STROBE Statement. http://strobe-statement.org/index.php?id=strobe-home. Accessed 14 Dec 2016.

[31] Ji C, Sykes L, Paul C, Dary O, Legetic B, Campbell NR (2012). Systematic review of studies comparing 24-hour and spot urine collections for estimating population salt intake. Rev Panam Salud Publica.

